# Intestinal Obstruction Following Appendicitis

**Published:** 1904-10-08

**Authors:** 


### INTESTINAL OBSTRUCTION FOLLOWING APPENDICITIS.

In hospital practice it is by no means uncommon for patients, who in reality are suffering from appendicitis, to be submitted to the surgeon as cases of intestinal obstruction. And this mistake in diagnosis is to some extent excusable when there is present a cessation of the passage of the bowel contents in the normal direction, due to inflammatory invasion and consequent paralysis of the walls of the intestine; though it must be admitted that the loss of activity in the bowel is occasionally enhanced by the premature administration of opium. It is not difficult to appreciate that the abdominal distension in such a case is a serious matter, and requires a certain display of therapeutic energy, but it will usually yield to turpentine enemata, and, if vomiting be absent, to the administration of strychnine by the mouth. But, in the abdomen as elsewhere, inflammatory exudation is apt to form fibrous tissue, which, by binding together adjacent coils of intestine, may seriously interfere with their peristaltic activities, and even cause complete intestinal obstruction. Or again, the same catastrophe may ensue from a loop of bowel, which has become fixed at its base by adhesions, becoming rotated, and so forming a volvulus. Obstruction may be caused, too, by obliteration of the lumen of the bowel, by contracting "bands" of fibrous tissue, or by strangulation of a loop of gut. That these dangers are not purely imaginary is shown in a paper by Dr. C. A. McWilliams [1] who has been able to collect and analyse reports of 86 cases of intestinal obstruction resulting from appendicitis. From a study of these he concludes that obstruction is most likely to follow when there has been abscess formation; this was so in 81 per cent. of his cases. In 10 per cent. no operation had been performed, and in 9 per cent. there had been an "interval" operation—*i.e.*, removal of the appendix after subsidence of an acute inflammatory attack. The onset of obstruction varied from within a few days of the appendix operation to seven years after. In all the cases where the seat of obstruction was mentioned it was in the small intestine. Dr. McWilliams' observations form one more indication for early operation in appendicitis, and in order that the chances of subsequent intestinal obstruction may be minimised, he advises that there should be as little handling of the intestines as possible, and that, whenever feasible, drainage should be avoided.

[1] Medical News, Sept 3.

